# Role of Macroscopic Image Enhancement in Diagnosis of Non-Muscle-Invasive Bladder Cancer: An Analytical Review

**DOI:** 10.3389/fsurg.2022.762027

**Published:** 2022-02-21

**Authors:** Prashant Motiram Mulawkar, Gyanendra Sharma, Ashwin Tamhankar, Utsav Shah, Rickaz Raheem

**Affiliations:** ^1^Department of Urology, Tirthankar Superspeciality Hospital, Akola, India; ^2^Tutor in Urology, University of Edinburgh, Edinburgh, United Kingdom; ^3^Chitale Clinics Private Ltd., Solapur, India; ^4^Apollo Hospital, Navi Mumbai, India; ^5^Sanjay Gandhi Post Graduate Institute of Medical Sciences, Lucknow, India; ^6^Milton Keynes University Hospital, Eaglestone, United Kingdom

**Keywords:** bladder cancer, image enhancement, fluorescence cystoscopy, narrow band imaging, image 1 S

## Abstract

Early diagnosis of non-muscle-invasive bladder cancer (NMIBC) is of paramount importance to prevent morbidity and mortality due to bladder cancer. Although white light imaging (WLI) cystoscopy has long been considered the gold standard in the diagnosis of bladder cancer, it can miss lesions in a substantial percentage of patients and is very likely to miss carcinoma *in situ* and dysplasia. Tumor margin detection by WLI can be inaccurate. Moreover, WLI could, sometimes, be inadequate in distinguishing inflammation and malignancy. To improve the diagnostic efficacy of cystoscopy, various optical image enhancement modalities have been studied. These image enhancement modalities have been classified as macroscopic, microscopic, or molecular. Photodynamic diagnosis (PDD), narrow band imaging (NBI), and Storz image 1 S enhancement (formerly known as SPIES) are macroscopic image enhancement modalities. A relevant search was performed for literature describing macroscopic image enhancement modalities like PDD, NBI, and image 1 S enhancement. The advantages, limitations, and usefulness of each of these in the diagnosis of bladder cancer were studied. Photodynamic diagnosis requires intravesical instillation of a photosensitizing agent and a special blue light cystoscope system. PDD has been shown to be more sensitive than WLI in the detection of bladder cancer. It is superior to WLI in the detection of flat lesions. Bladder tumor resection (TURBT) by PDD results in more complete resection and reduced recurrence rates. PDD-guided TURBT may have some role in reducing the risk of progression. Narrow band imaging provides increased contrast between normal and abnormal tissues based on neovascularization, thereby augmenting WLI. NBI requires a special light source. There is no need for intravesical contrast instillation. NBI is superior to WLI in the detection of bladder cancer. The addition of NBI to WLI improves the detection of flat lesions like carcinoma *in situ*. NBI is not useful in predicting invasive tumors or grades of tumors. NBI-directed TURBT reduces recurrence rates and recurrence free survival. But its efficacy in retarding progression is unproven. Image 1 S-enhancement utilizes software-based image enhancement modes without the need for a special light source or intravesical contrast instillation. This system provides high-quality images and identifies additional abnormal-looking areas. Another advantage of this system is simultaneous side-by-side visualization of WLI and enhanced image, providing WLI images as the control for comparison. As with PDD, S-enhancement produces a lower rate of a missed bladder cancer diagnosis. The system significantly improves the diagnosis of NMIBC. The sensitivity and negative predictive value of image 1 S enhancement increase with the increase in cancer grade. A negative test by S-enhancement effectively rules out NMIBC. All the image enhancement modalities have proven their utility in improving detection and short-term cancer control. But none of these modalities have proven their utility in delaying progression, or in long-term cancer control. Cancer progression and long-term control are governed by the biological nature of cancer cells. Early detection by optical enhancement may not be of utility in this regard. Well-designed studies are needed to establish the efficacy of these modalities in the evaluation of patients with bladder cancer. The last word, in this regard, is yet to be written.

## Introduction

Among urological cancers, non-muscle-invasive bladder cancer (NMIBC) has a better prognosis. But bladder cancer, as a whole, is the costliest solid tumor to treat (1). Although cytologic analysis, biomarkers, and imaging are useful tests, the mainstay of diagnosis rests on cystoscopy (1). White light imaging (WLI) cystoscopy, the most commonly utilized diagnostic modality (2), has been at the forefront of the diagnosis of bladder cancer for more than a century. WLI is the “Gold standard” for the diagnosis of bladder cancer (1, 3). Once detected, a lesion is managed by transurethral resection (TURBT) to know its histologic grade and stage. Around three quarters of patients are diagnosed by WLI and resection (2). Conventional cystoscopy can miss around 25% of bladder tumors (4). The technique of TURBT can be piecemeal or *en bloc*, and there is an important concern that new enhanced optical techniques can affect the *en bloc* resection of bladder tumors (5). However, the technique of resection during TURBT is not being discussed in this review.

The quality and adequacy of resection vary among institutions and from surgeon to surgeon. The risk of recurrence cannot be explained by the biology of a tumor alone (6). Possible mechanisms of recurrence are incomplete resection of tumors (overlooked residual tumors), microscopic residual tumors (regrowth of tumors), implantation of tumor cells in raw sites, and new occurrence of tumors (7). At least two of these mechanisms direct toward incomplete TURBT (8). Incomplete TURBT, especially in high-grade tumors, will negatively affect the prognosis of bladder cancer. Cystoscopy can also miss occult neoplastic changes in flat urothelial. Lesions in this category would be dysplasia, low- and high-grade intraurothelial neoplasia, and carcinoma *in situ* (CIS) (9). It is a well-known fact that WLI can miss some lesions. Such missed lesions can show up as “recurrences” on check cystoscopies. It is not technically possible to perform a perfect “text book TURBT” like complete macroscopic clearance, thorough resection of a tumor base, and separate resection of tumor margins. An incomplete TURBT, even if inadvertent, may change the prognosis of illness in a given patient. The risk of residual tumors is acceptably low at around 10% in Ta disease, but in T1 disease it could be as high as 62% (7). This residual tumor, labeled as recurrence in T1, is supposed to be a poor prognostic factor.

If not the biology, then other factors like a machine or humans behind the machine might be a factor influencing the completeness of TURBT. In this review, we talk about the machine. To improve the diagnosis of suspicious bladder lesions, optical enhancement modalities are used. This analytical review is about the role of macroscopic image enhancement modalities in the management of NMIBC.

## Materials and Methods

A PubMed search was performed with the following criteria: (bladder cancer) and (narrow band imaging); (Bladder cancer) and (“Fluorescence Cystoscopy”); (bladder cancer) and (SPIES); (bladder cancer) and (image 1S); (bladder cancer) and (optical enhancements). Initially, titles were screened to identify eligible articles followed by the screening of abstracts. Finally, full-text articles were read. In addition, individual searches were performed on Google Scholar, Scopus, ScienceDirect, and SpringerLink. Reference lists of the selected articles were also searched, and additional studies were included. All authors participated in the formulation of the search strategy and selection of the articles. There were no specific exclusion criteria. Editorials, reviews, opinions, debates, and letters to editors were also included if found relevant. There was no particular time frame applied for the literature. The advantages, limitations, and usefulness of different image enhancement modalities in the diagnosis and management of bladder cancer were studied. A total of 387 articles were retrieved; of which 127 were included and 260 were rejected. Most of the literature was combined in a simple narrative fashion.

## Results and Discussion

### Classifications of Image Enhancement Technologies

Image enhancement technologies are usually classified as macroscopic or microscopic.

#### Macroscopic Techniques

Macroscopic technologies are also called “wide field view.” In this, a surgeon diagnoses a lesion looking at mucosal morphology, color, and vessels. Macroscopic imaging identifies ulcers, erosions, papillary lesions, reddish or white spots, and the presence of vessels in the mucosa and neo-angiogenesis (10). Macroscopic techniques are further classified as wide-field WLI or contrast enhancement techniques.

##### Wide-Field WLI

High-definition camera systems (1,080 p) provide a >5-fold increase in image resolution. In addition, image filtering and zooming improve picture quality by more than 30% (11). Camara systems with a resolution higher than high definition are being used nowadays. Newer systems with 4 and 8K (UHD) are being used nowadays. Wide-field endoscopy also uses enhancement modalities like close focus, dual focus, optical zoom, and electronic zoom to gain better vision and have a low rate of missed lesions (10). Data on the impact of wide-field WLI on improved diagnosis and better outcomes in bladder cancer is awaited. Wide-field WLI, as a technique, is not part of this review.

##### Contrast Enhancement Techniques

*Virtual Chromoendoscopy.* Virtual chromoendoscopy improves the detection of lesions by changing spatial variance and scattering properties. Herein, either incident light is changed to selected waveforms or images generated after illumination are processed to transform color or change color tone, or change the contrast. Based on the stage of processing of light, VCE is further classified into three categories: pre-processing, post-processing, pre- and post-processing (10).

Commonly used pre-processing VCE techniques are narrow-band imaging (NBI), red dichromatic imaging, and blue light imaging. In pre-processing VCE, a modified spectrum of light is switched on, and it results in color transformation depending on the peak absorption of hemoglobin and different tissue elements. In post-processing VCE, incident light is white light, but processing occurs at the camera processor level by color transformation and color tone change to produce an effect similar to that of pre-processing VCE. Commonly used post-processing VCE techniques are Fujinon intelligent chromoendoscopy (FICE) and image 1 S (previously called Storz professional image enhancement system or SPIES). FICE is commonly used in gastroenterology but is not discussed further.

*Fluorescence Endoscopy.* This technique utilizes different fluorophores. A fluorophore is a fluorescent chemical compound that, on excitation with light, reemits light. Fluorescence signals emitted by fluorophores are dependent on wavelength. There are special optical fibers used in this system that block the false excitation of light and capture only a part of the emitted light, thereby producing a final image (10). Commonly used fluorescence endoscopy techniques are autofluorescence imaging (AFI), near-infrared imaging (NIR) with indocyanine green (ICG), and photodynamic diagnosis (PDD). AFI is commonly performed in gastroenterology. Urological applications of NIR and ICG include tumor localization, selective arterial clamping during partial nephrectomy, mapping of lymph nodes during radical prostatectomy, radical cystectomy and penile cancer, urethral viability assessment during urethroplasty and varicocelectomy (12, 13). NIR and ICG are not being discussed further.

#### Microscopic Techniques

These techniques visualize microstructural details of tissues and act like an optical biopsy, which acts like *in vivo* tissue biopsy. Confocal laser endomicroscopy (CLE) is a promising tool for the optical diagnosis of bladder cancer. Microscopic techniques are not part of this review.

### Fluorescence Cystoscopy/PDD

#### Principle

The basic principle behind PDD is the selective emission of fluorescence by cancer cells. Commonly used substances are 5-aminolevulinic acid (ALA) and hexaminolevulinate (HAL). When instilled in the bladder, these are preferentially taken up by cells with high metabolic turnover. Drugs are incorporated in cellular hem biosynthesis metabolism, wherein prodrugs are converted to protoporphyrin IX (PpIX). Pathological and normal tissues have different concentrations of fluorescence molecules. These molecules are excited by light of an appropriate wavelength. After molecules relax to the ground state, they emit photons. Excitation photons have more energy than fluorescence photons. If the energy of light is more, the wavelength is short and vice versa. Hence, the light that is emitted from a fluorescence photon will have a longer wavelength than the illuminating photon. Hence, it is possible to discriminate between two types of light by PDD (14).

#### Technical Consideration

##### Equipment

A high-performance light source called Karl Storz D-Light C is used for PDD (15). The light source has a band pass filter, which produces blue light of 360–450-nm wavelength. This light source can be switched from white light to fluorescence mode with a footswitch. Rigid telescopes with 0-, 12-, 30-, and 70-degree vision are commonly used. Flexible cystoscopes and chip-on-tip video cystoscopes are also available (15, 16). Apart from Karl Storz, Germany, Richard Wolf, Germany also makes PDD systems (17).

##### Photosensitizing Agents

Typically, 5-ALA is used as a freshly prepared solution of 1.5 gm 5-aminolevulinic acid dissolved in 50 ml 5.7% sodium monohydrogenphosphate. Dwell time of 2–3 h is recommended (18). ALA yields high sensitivity especially for the diagnosis of CIS and pinpoint lesions. But the amount of ALA entering cancerous cells is limited, as the cellular uptake of ALA is limited due to ionic structure. To overcome this shortcoming, an increased dose or increased dwell time is needed (19).

In contrast, the ester form of a drug penetrates cells better. Once inside a cell, the ester form is hydrolyzed back to ALA by tissue esterases. Lange et al. (19) are credited for publishing initial experiences in using 5-aminolaevulinic acid hexylester hydrochloride (HAL)-induced PDD. Advantages of HAL are better solubility in urine and water, higher local bioavailability, more PpIX formation at lower doses, homogenous fluorescence, less vulnerability to photobleaching, and excellent florescence intensity (20). Currently, HAL is available as Cysview (Photocure US, Princeton, NJ, United States) or Hexvix (Photocure ASA, 0275 Oslo, Norway) as a kit containing 100 mg hexaminolevulinate HCL powder and 50 ml phosphate-buffered saline as a diluent. It can be stored for 2 h at 2–8°C. The solution is usually prepared once a patient is admitted. Once the patient is ready, a catheter is passed. All urine in the bladder is emptied, and a freshly prepared HAL solution is instilled slowly in the bladder. The usual dwell time is 1–3 h. HAL should not be used with silver-coated catheters (15).

Intravesical HAL is usually well-tolerated. Common but not clinically significant adverse events reported are bladder spasm, dysuria, and hematuria (15). HAL is contraindicated in patients with a history of known allergy to HAL or a history of porphyria. There has been one case report of anaphylaxis after HAL or PDD (21).

Hexaminolevulinate can be used by the intravesical instillation route only. 5-ALA can also be used by intravesical and oral routes. A comparative randomized multicentre phase II/III study on oral 5-ALA in doses of 10 and 20 mg/kg was reported by Inoue et al. (22). The authors reported a higher rate of tumor detection with the 20 mg/kg dose and suggested this dose for clinical use (22). Nakai reported the diagnostic efficacy, safety, and tolerability of PDD with oral 5-ALA in a multicentre phase III trial. The dose used was 20 mg/kg of oral 5-ALA (23).

Hypericin, a plant derivative, is another fluorophore used in urology. Decreased susceptibility to photobleaching and better specificity are possible advantages of hypericin. But low solubility is an issue. Efforts to improve its solubility using solvents like polyvinylpyrrolidone and albumin are being investigated. Hypericin is well-tolerated, and its fluorescence is maintained up to 16 h (24). In a study to determine the optimum dose of hypericin, 225 ug for 30 min was concluded to be the optimum dose for intravesical instillation (25). In addition, pirarubicin has also been used for PDD (26).

##### PDD Cystoscopy Procedure

After a drug dwell time of 1 h, a patient is taken to an operating room. Rigid cystoscopy is performed. Initially, systematic WLI is performed, and lesions are marked in bladder map proforma. Then, the blue light is switched on, Initially, the scope is kept at the bladder neck to identify tangential artifacts. This ensures that the photosensitizing agent is applied properly (15). For a detailed evaluation of a lesion, the scope should be close and as perpendicular as possible to the bladder wall or a tangential artifact may occur. As the observation angle becomes more tangent, the bladder mucosa appears more fluorescent (27).

Lesions are seen as bright red fluorescence. The dark blue background illumination in PDD hampers proper depth perception. Hence, a biopsy is usually performed with white light. Similarly, resection is also usually performed with white light. Urine fluoresces green under PDD. Therefore, it is advisable to empty it periodically (15). It is advisable to perform a mapping cystoscopy initially before starting the resection as fluorescence fades with time.

##### The Learning Curve of PDD

Gravas et al. evaluated the learning curve of PDD with HAL (28). The authors compared the agreement statistics among cystoscopies performed by senior residents and experienced urologists. After an experience of 20 cases, inexperienced observers had good agreement with the experienced ones, which improved to an excellent agreement after 30 cases, suggesting a learning curve of around 20–30 cases (28). The experience of a urologist performing PDD is an important factor for the quality of PDD (29). An experienced urologist who takes more biopsies is likely to yield more false positives and resect more tumors. As the learning curve of urologists goes beyond 12–18 months, the number of false positives goes down (29).

#### Performance of PDD: Diagnosis, Residual Tumors, Short-Term Recurrence, and Effects on Treatment

Zaak et al. are credited for one of the first few clinical data on the role of PDD with ALA in bladder cancer. The authors found that PDD was more efficient than WLI and urine cytology in diagnosing high-risk urothelial lesions (18). In initial studies, PDD was performed using 5-ALA and HAL. Both the fluorescence agents have shown improved sensitivity but poor specificity (Table 1).

**Table 1:** Characteristics of sensitivity and specificity of photodynamic diagnosis (PDD) and white light imaging (WLI).

**References**	**Photosensitiser**	**PDD sensitivity**	**PDD specificity**	**WLI sensitivity**	**WLI specificity**
Kriegmair et al. (30)	5ALA	97	67	73	69
Riedl et al. (4)	5ALA	95	43	76	*
Filbeck et al. (31)	5ALA	96	67	68	66
Koenig et al. (32)	5ALA	87	59	69	89
Jichlinski et al. (33)	HAL	96	43	73	43
Schmidbauer et al. (34)	HAL	97	*	78	*

*No data.

Hexaminolevulinate is the most commonly used agent in PDD, and literature regarding HAL is reviewed preferentially. Grossman et al. reported their experiences in a multicentric trial on PDD using HAL (35). The authors found that PDD was efficacious by detecting at least one more Ta lesion than WLI in 29% of patients and one more T1 lesion in 15% of patients. WLI detected at least one more Ta lesion than PDD in 9% of patients and one more T1 lesion in 5% of patients (35). Mean detection rates for PDD and WLI were 95 and 83% for Ta (*p* = 0.001) and 95 and 86% for T1 lesions, respectively. Interestingly, all additional lesions detected by PDD in this study were grade 3.

Stenzl et al. published a prospective randomized trial studying the effects of improved detection of bladder cancer on early recurrence rates (36). The authors recruited patients with an increased risk of recurrences like more than one initial or recurrent tumor and recurrence within 1 year of previous bladder cancer. These patients were randomized to TURBT with WLI or PDD using HAL. Recurrence rates were 56% in WLI and 47% in the PDD groups (*p* = 0.026). There was a 16% relative reduction in the risk of recurrence (36).

Geavlete et al. reported recurrence rates of 23.2% with WLI and 5.3% with PDD in a HAL group in over 18 weeks follow-up (37). In another study, the same group reported their experiences in a single institute prospective randomized trial comparing TURBT with WLI or HAL PDD (38). Tumor detection rates were better in the PDD arm. Three-month recurrence rates were 7.2 vs. 15.8% in PDD vs. WLI arm. One-year recurrence rates were 21.6 vs. 32.5% respectively. Two-year recurrence rates were 31.2 and 45.6% (38). Fradet et al. published results of phase III multicentre trial wherein WLI was compared with PDD using HAL (39). This study was mainly directed toward the detection of CIS. Fifty-eight out of a total of 196 patients had CIS lesions. A total of 113 lesions were detected in these 58 patients. Of these, 92% of the lesions were detected by PDD and 68% by WLI, but 5 (of 113) CIS lesions were detected only by random biopsy of normal-looking mucosa. Although tumor detection rates were higher in the PDD group, there were some, albeit scant, numbers of lesions detected only by WLI.

Schmidbauer et al. reported a within-patient comparison of WLI and PDD with HAL (34). The study was designed to evaluate the detection of CIS. In this study, 83 out of 211 patients had CIS. Among these, 22% were detected by PDD alone, 75% of CIS lesions were detected by WLI as well as PDD, and 2% of lesions were detected by WLI alone and 1% by non-guided biopsy. A lesion detected by non-guided biopsy was located in the prostatic urethra where PDD is less discriminating because of tangential artifact (34). PDD cystoscopy detected a significantly higher number of additional CIS lesions than WLI. Interestingly, in this study, false positive rates for PDD were similar to those for WLI cystoscopy (13 vs. 10%). PDD cystoscopy detected 28% more patients with CIS lesions than WLI cystoscopy (34). Blanco et al. evaluated the performance of HAL PDD in the detection of urothelial lesions in patients with a high risk of progression (40). The study also included patients who have received BCG. HAL PDD had 90.1% sensitivity and 87.5% specificity. The positive predictive value was 95.2%, and the negative predictive value was 77.8% (40).

There are some doubts on whether additional detection of few more papillary tumors has any impact on treatment planning. Filbeck et al. reported a within-patient evaluation to see if the addition of PDD with 5ALA during TURBT would lead to therapeutic consequences (41). In this study, the addition of PDD led to the detection of additional tumors missed by WLI in 5.1% of patients, and the gain of additional information in 15.3% of patients leading to a change in treatment strategy in 9% of the patients. Jocham et al. evaluated whether the improvement in detection by PDD had an impact on treatment planning (42). This was an open, comparative, within-patient study. Anonymized data from patients were sent to a blinded urologist, and he was asked to formulate a treatment plan as per European urology association bladder cancer guidelines. The diagnosis with PDD led to the recommendation of additional treatments like BCG and topical or prophylactic chemotherapy, TURBT, and cystectomy. In two patients where cystectomy was advised after PDD imaging, no tumor was detected on WLI. TURBT with PDD resulted in improved treatment decisions in a significant number of patients (*p* < 0.0001) (42).

The role of HAL PDD in the screening of men aged 60–70 years with microscopic hematuria and positive bladder tumor marker was evaluated by Hedelin et al. (43). The authors reported that HAL PDD in this screening scenario was not useful. Rather, they suggested HAL PDD cystoscopy in older male smokers with >25 RBC/ul (43).

A prospective phase II study on PDD using a flexible cystoscope and rigid cystoscope for diagnosis of bladder tumors found PDD with flexible cystoscope to be slightly inferior to PDD with rigid cystoscope (44). On the contrary, in a recent study, Drejer and colleagues highlighted the beneficial effects of flexible cystoscopy in post TURBT follow up. A reduction by 33% was noted in the short term recurrence rates over a median follow-up of eight months (45).

Mowatt et al. from Aberdeen Technology Assessment Review Group reported a meta-analysis of 27 studies including 2,949 patients (46). In patient-level analysis, PDD yielded 92% sensitivity (95% CI 80–100%); on the contrary, WLI had 71% sensitivity (95% CI 49–93%). But the specificity of PDD was lower at 57% (95% CI 36–79%), whereas the specificity of WLI was 72% (95% CI 47–96%). In biopsy-level analysis, PDD had a sensitivity of 93% (95% CI 90–96%); WLI had a sensitivity of 65% (95% CI 55–74%). The specificity of PDD was 60% (95% CI 49–71%). The specificity of WLI was 81% (95% CI 73–90%). Considering 5-ALA and HAL separately (46) in patient-based detection, the median sensitivity and specificity of 5-ALA were 96% (64–100%) and 52% (33–67%), respectively; whereas for HAL sensitivity was 90% (53–96%) and specificity was 81% (43–100%). For biopsy-based detection, sensitivity and specificity were 95% (87–98%) and 57% (32–67%) for 5-ALA, and 85% (76–94%) and 80% (58–100%) for HAL. Thus, the sensitivity of PDD was higher, but specificity was lower than that of WLI. For detection of lower risk and less aggressive tumors, the sensitivity of PDD was comparable to that of WLI in patient-level detection but higher in biopsy-level detection. For detection of high-risk and more aggressive tumors including CIS, the sensitivity of PDD was better in patient-level as well as biopsy-level detection. In this meta-analysis, PDD was found to be more sensitive but less specific than WLI both at the patient level and biopsy level. This advantage of PDD was more pronounced in the detection of more aggressive and high-grade tumors including CIS.

Mowatt et al. reported a meta-analysis comparing WLI with PDD (46). In post TURBT repeat cystoscopies done between 10–14 days and 10–15 weeks, the overall (both WLI and PDD combined) relative risk of pTa and pT1 residual tumors was 0.37 (95% CI 0.20–0.69). Residual tumors in the WLI group were RR 0.32 (95% CI 0.15–0.7), and in the PDD group, RR was 0.26 (95% CI 0.12–0.57). The PDD-guided TURBT was beneficial in terms of residual tumor rates. In the Mowatt meta-analysis (46), two studies reported outcomes of recurrence-free survival. The benefit of recurrence-free survival in favor of PDD was observed at 24 months but not at 12 months.

However, the beneficial effects of PDD in recurrence are not universal across all studies. A Swedish multicenter prospective randomized study on PDD using 5-ALA compared with WLI was reported by Schumacher (47). Patients were randomized to TURBT with WLI and TURBT with PDD. All follow-up cystoscopies were performed under WLI. The authors did not find any advantage of PDD over WLI in the form of recurrence-free and progression-free survival rates. However, more lesions were detected in the PDD group on the within-patient comparison. The results of this study have been criticized for inexperienced observers and mean exposure time of 5-ALA of only 2.1 h (48). A similar multicenter prospective placebo-controlled randomized trial was reported by Stenz et al. (49). In this trial, the tumor detection rate was higher in the PDD group than in the placebo group. However, the recurrence-free and progression-free survival rates were similar in the PDD and placebo groups.

A systematic review and meta-analysis by Shen et al. assessed the diagnostic accuracy and therapeutic efficacy of TURBT with WLI or PDD (50). In eight studies the comparator was 5-ALA, in 3 studies HAL, and 2 studies HAL and 5ALA; in one study, no record of fluorescence agent was available. Unexpectedly, this meta-analysis did not find PDD to be superior to WLI in tumor detection and CIS detection rates. Residual tumor rates were, however, higher in the WLI group. Short-term recurrence-free survival and progression-free survival were also not different in the WLI and PDD groups (50).

Burger et al. reported a meta-analysis containing raw data from prospective studies on 1,345 patients (51). WLI and PDD HAL were the comparators. PDD detected significantly more Ta [14.7%; *p* < 0.001; odds ratio (OR): 4.898; 95% CI, 1.937–12.39] tumors as well as CIS (40.8%; *p* < 0.001; OR: 12.372; 95% CI, 6.343–24.133) lesions than WLI. By PDD, at least one additional Ta or T1 lesion was detected in a quarter of the patients. Similarly, in a quarter of the patients, CIS was detected only by PDD. This improvement in detection was seen in primary recurrent cancers as well as patients with high and intermediate risks. One-year recurrence rates were lower in PDD than in WLI [34.5 vs. 45.4%, *p* = 0.006; RR 0.761 (0.627–0.924)]. This benefit was seen in Ta, T1, and CIS lesions and high-risk and low-risk groups (51). Similar outcomes were reported in a meta-analysis reported by Kausch et al. (52).

##### The Issue of False Positives, Post BCG PDD, and Other Limitations of PDD

One of the disadvantages of PDD is high false positive rates (1–26%) (53). Common causes of false positives during PDD cystoscopy are erythema, bladder inflammation, trauma due to cystoscope, prior intravesical therapy, squamous metaplasia, and scar from old resection site. Reasons for variable specificities of PDD across different studies are different dwell times of photosensitizing agent, the experience of urologist, differences in techniques and equipment, and differences in patient population and pathologic classification system used (27). With regard to false positives, HAL performs better than 5-ALA (46).

Photodynamic diagnosis is usually avoided within 3 months of intravesical BCG therapy because more than one BCG instillation during this period has a significant effect on false positive rates of PDD (54). Grimbergen et al. evaluated the effect of previous intravesical therapy (BCG, mitomycin, or epirubicin) on false positive rates (27). Patients were recruited in three groups; intravesical therapy within the prior 6 months, more than 6 months, and no intravesical therapy. False positive rates were 39.6, 30.6, and 25.7% (*p* <0.025) (27). In this study, around one-third of lesions were overlooked by WLI, and one-third of these lesions were high-risk lesions. These high-risk lesions are likely to affect patient outcomes significantly. Therefore, the authors have recommended PDD shortly even after intravesical therapy (27).

Resection of all visible fluorescence mucosae yields better progression-free survival. It is possible that these false positive lesions may be premalignant. The role of p53 and p16 immunoreactivity in PDD false positive lesions, thinking these to be premalignant, was evaluated by Hendricksen et al., but little evidence for these lesions being premalignant was found (55). Matsuyama et al. determined genetic instabilities in the bladder mucosa suspected to be having CIS by 5-ALA-based cystoscopy (56). They found substantial early genetic changes in chromosome 9 in non-malignant fluorescence cells (56). As most of the studies did not perform random biopsies, the false negatives with PDD should be interpreted with caution. But most so-called false negatives are detected by WLI and should not affect the patient outcome (53). The optimum dwell time for HAL is 60 min. Patients with urgency may not retain the drug that long and may lead to reduced uptake. Improper maintenance of equipment, such as mixing the PDD equipment with other cystoscopy equipment, may be a source of error. Doing TURBT under blue light is technically challenging. Therefore, most surgeons do it with WLI. White light causes rapid elimination of PpIX, leading to photobleaching. This may lead to missed tumors during TURBT especially if it is prolonged. Apart from these, the limitations of PDD are minimal (53).

#### Long-Term Effects: Effects on Long-Term Recurrence and Progression, and Effect on Post-radical Cystectomy Outcome

Stenzl et al., in their multicentric prospective randomized trial on cystoscopy and TURBT with WLI and PDD using HAL, reported improved detection, but a subsequent decrease in disease progression was not found (36). In the meta-analysis of Mowatt et al. (46), the benefits of using PDD TURBT favored PDD but were not statistically significant in the long run. The along-term study showed trends toward lower cystectomy rates in patients who underwent PDD TURBT (48).

Babjuk et al. from the Czech Republic reported their data-randomized trial studying long-term follow-up of patients undergoing TURBT with WLI or PDD. The mean follow-up in WLI was 20.7 months and 22.4 in the PDD group. At 1 and 2 years, recurrence-free survival was 39 and 28% in the WLI group, whereas it was 66 and 40%, respectively, in the PDD group (*p* = 0.008) (8). The beneficial effect of TURBT with PDD was significantly higher on multiple and recurrent tumors. Similarly, the beneficial effect was more evident at 1 year than 2 years (8). Another set of long-term follow-up data was presented by Daniltchenko et al. (57). A total of 102 patients were randomized to TURBT with WLI or PDD with 5-ALA. Study groups were similar except that more patients with solitary tumors were randomized to TURBT with WLI. Median follow-up in the WLI and PDD groups was 39 and 42 months, respectively. The median time to the first recurrence was 5 and 12 months. Recurrence rates at 2, 12, 36, and 60 months were 41, 61, 73, and 75% on the WLI group, whereas it was 16, 43, 59, and 59% in the PDD group. Among the 51 patients in each arm, progression occurred in 9 and 4 patients, respectively (57).

Grossman et al. reported long-term data on the use of PDD during TURBT (48). This is an extension of their initial pivotal phase III study (36). Patients were followed up for a median period of 53 and 55.1 months in the WLI and PDD groups, respectively (48). Intravesical therapy rates were comparable between the WLI and PDD groups. In the WLI group, 31.8% of patients remained tumor-free, whereas 38% remained tumor-free in the PDD group. In the WLI group, the median time to recurrence was 9.4 months compared with 16.4 months in the PDD group (*p* = 0.04). Cystectomy rates were lower in the PDD group than in the WLI s at 4.8 and 7.9%, respectively (*p* = 0.16). This may indicate a trend toward improved bladder preservation in the PDD group (48).

Denzinger et al. published their data on recurrence rates with an 8-year follow-up (58). In this study, 5-ALA was used as the photosensitizing agent. A total of 301 patients were recruited; of them, 191 were evaluable for efficacy. The patients were followed up for a median period of 83 and 86 months in the WLI and PDD arms, respectively. In the WLI group, recurrence-free survival was 73, 64, 54, and 45% at 2-, 4-, 6-, and 8-year follow-up; for the PDD group it was 88, 84, 79, and 71%, respectively, favoring TURBT with PDD (*p* = 0.0003). The beneficial effect of PDD was maintained across all prognostic groups irrespective of the use of intravesical therapy. In this study, the authors, however, did not find a significant beneficial effect of PDD TURBT on preventing progression to the muscle-invasive stage (58).

The impact of PDD TURBT on oncologic outcome after radical cystectomy was reported by Gakis et al. in a retrospective study (59). The authors retrospectively evaluated the effect of PDD (ALA or HAL)- guided TURBT on the outcome of 243 consecutive radical cystectomies. In univariate analysis, the PDD TURBT group had a higher median number of TURBTs before radical cystectomy, a higher number of re-resections, more frequent BCG treatment, and longer intervals between first TURBT and radical cystectomy as well as a lower rate of adjuvant chemotherapy. Three-year recurrence-free survival, cancer-specific survival, and overall survival after TURBT were significantly better in the PDD with HAL TURBT group. In addition to the pathologic tumor stage, nodal stage, and surgical margins, the performance of TURBT with HAL guide was an independent predictor of recurrence, cancer-specific death, and overall death after radical cystectomy (59). HAL-guided TURBT was found to be an important predictor of improved survival after radical cystectomy for the first time in this study (59). In contrast, May et al. in their multi-institutional retrospective study, did not find a significant impact of PDD during TURBT on prognosis after radical cystectomy (60).

Yuan et al. reported a meta-analysis of 12 RCTs including 2,258 patients. PDD TURBT had lower recurrence rates, delayed recurrence, and improved recurrence-free survival at 1 and 2 years (61). Nine studies in this meta-analysis reported data on progression to the muscle-invasive stage. No significant difference was found between WLI and PDD in progression rates (61).

The definition of progression in studies has been imprecise and inconsistent. Various definitions indicating progression by different investigators are development of muscle-invasive (T2) disease, development of CIS, uncontrollable CIS, nodal metastases, distant metastasis, increase in tumor stage, increase in tumor grade, and disease-worsening requiring treatment change like the need for cystectomy, radiation therapy, or systemic chemotherapy (62). Because of some of these imprecise definitions of progressions, statistically significant progression endpoints have not been achieved in some bladder cancer trials. This issue was discussed in detail by the International Bladder Cancer Group (IBCG). The authors suggested new definitions of NMIBC progression, namely, “an increase in T stage from CIS or Ta to T1 (lamina propria invasion), development of T2 or greater or lymph node (N+) disease or distant metastasis (M1), or an increase in grade from low to high” (62). In view of these new definitions of progression, Kamat et al. reanalyzed their previously published data (36). In the reanalysis (63), TURBT with PDD showed a trend toward lower progression in PDD compared to WLI. The new definitions identified more patients at risk of progression in the WLI and PDD groups. Time to progression was significantly prolonged in the PDD group *vis a vis* the WLI group. A lower rate of progression was particularly evident in progression from Ta to CIS. This is important, as detection rates for CIS are quite poor in WLI. Improved progression rates in the PDD group were unrelated to intravesical BCG treatment, as the BCG rates in both groups were comparable (63).

#### Cost-Effectiveness

High recurrence rate, low mortality rate, and need for long-term follow-up make bladder tumors the costliest to treat. PDD is beneficial in cost savings. Although there is an initial cost in acquiring new equipment and recurrent cost of HAL, reduced recurrence rates, reduced need for intravesical instillations, and less intensive follow-up regimen lead to net savings of GBP 45,500 per 100 new patients with NMIBC (64). Daniltchenko et al. performed a cost analysis of 5-ALA-based TURBT based on several procedures required, hospitalization, cost of ALA, and equipment amortization. The authors reported savings of USD 425 per patient per year with PDD (57). In a similar analysis with a median follow-up of 7 years, Burger et al. reported savings of EUR 168 per patient per year with PDD TURBT (65). Malmström et al. through a decision analytical model, projected that using HAL PDD for all first TURBTs and all recurrence TURBTs in 1 year would lead to net savings of SEK 1321716 to the Swedish health exchequer (66). Garfield et al. (67), in their cost-effectiveness study on HAL PDD using a probabilistic decision tree model, reported reduced costs with the use of PDD compared to WLI alone ($25,921 with PDD vs. $30,581 with WLI) (67). Using PDD throughout the management of NMIBC leads to significant cost savings and improved quality-adjusted life years (68).

#### PDD: Current Place

Photodynamic diagnosis is the most extensively studied macroscopic image enhancement technique. PDD-directed TURBT leads to more complete TURBT and reduced recurrence rates, and improved recurrence-free survival. It decreases recurrence rates more so in patients with high-risk lesions. However, most data on progression use an imprecise definition of progression. Reanalysis of data with a new precise progression definition suggests beneficial effects of TURBT with PDD on delaying progression. PDD may have a beneficial effect on post-radical cystectomy outcomes.

It is still not used widely because of the expensive equipment and recurring cost of a fluorescence agent. One survey from German-speaking countries reported that WLI was only performed by 60.2% of practitioners, with additional PDD performed by 36.8% (69). In a large Chinese study comprising 14,260 patients, around 74.3% of the patients were diagnosed by WLI alone. PDD had an additional detection rate of 1% (2). Moreover, its low specificity leads to additional biopsies and increased anxieties. Also, it is not yet available in many countries (14).

### Narrow-Band Imaging

#### Principle

Narrow-band imaging is a high-resolution endoscopy technique that filters out the red spectrum of light and selectively transmits blue (415 nm) and green (540 nm) segments of white light without actually utilizing any intravesical contrast medium (1, 3, 10). As the blue and green spectra coincide with absorption peaks of hemoglobin (oxy-hemoglobin: 415, 542, 577 nm and deoxy-hemoglobin: 430 and 555 nm), tissues rich in hemoglobin content will have a different appearance compared to adjacent tissues. This contrast helps clinicians to predict the possibility of neovascularized malignant tissue in a better manner than conventional white light cystoscopy. Mucosal capillaries are accentuated by the blue segment (415 nm), and relatively deeper located submucosal capillaries appear cyan by the green segment (540 nm) of filtered light.

#### Technical Consideration

Narrow-band imaging is available either as an integrated video cystoscope or a camera head that can be fitted to a telescope. There is no need to instill an exogenous contrast. The NBI mode would highlight the microvasculature to identify neovascularisation and would enable the clinician to find out about the boundary between vascularized and non-vascularised structures.

Narrow-band imaging provides a better view of the urothelium. The vasculature appears dark green to black. The normal mucosa looks pale white (70). The contrast between flat growths and normal mucosa is enhanced, leading to improved diagnosis. It sometimes becomes difficult to distinguish between flat and papillary lesions with NBI (71). As expected, NBI produces suboptimal images in the presence of hematuria and inflammation (72, 73). Moreover, it should always be combined with WLI cystoscopy to avoid false positives (73). NBI is useful not only in judging margins of resection but also in rapidly identifying lesions that could have been missed on WLI. Carcinoma *in situ*, an aggressive variety of urothelial cancer, would certainly be seen in an efficient manner because of a characteristic appearance based on higher vascularity.

Initial primary versions of NBI scopes had relatively lesser illumination than white light, making it difficult to be used as the primary method of diagnosis; however, in 2013, second-generation NBI scopes (EVIS LUCERA ELITE and EVIS EXERA III, Olympus) were manufactured and had high illumination intensity (10). In the third-generation NBI system (EVIS X1), Olympus, Japan designed two LED lights with specific central wavelengths of 460 and 540 nm to provide a narrow bandwidth (10). This modification helped increase the longevity of the light source while reducing the overall volume of the system. NBI technology is now incorporated into flexible scopes as well for pure diagnostic office-based procedures to pick up recurrences and flat lesions in patients under regular surveillance for bladder cancer (1).

##### Learning Curve

There is no significant learning curve with NBI. Bryan et al. reported on their experiences in NBI being performed by a “new user” (71). A trainee only underwent one session of training and observation of the technique. In this series, the new user detected 0.65 additional lesions with NBI as compared with WLI (71). This is not statistically different than the number of lesions detected by an experienced user (74). In another study on evaluation of 50 cystoscopy images by WLI and NBI shown to independent observers, minimal individual variation was seen among the observers. Detection rates between experienced users and novice were not different (75). This suggests that NBI cystoscopy is easy to learn and master.

#### Role in Diagnosis

The first clinical experience in performing NBI cystoscopy was published by Bryan et al. (74). In this study, the authors performed NBI flexible cystoscopy on 29 patients diagnosed as having recurrent bladder cancer that has been previously diagnosed by WLI. The NBI examination detected a mean of 0.52 additional tumors in this small cohort. These additional tumors might have been labeled “early recurrence” on subsequent WLI cystoscopy. However, this study did not study CIS lesions. Herr and Donat reported their diagnostic cohort study on WLI vs. NBI (70). The authors first performed WLI cystoscopy and then NBI cystoscopy on patients with suspected recurrent NMIBC. NBI was able to diagnose more lesions than WLI, more so CIS. WLI detected 2.3 lesions per patient, whereas NBI detected a mean of 3.4 lesions in this cohort (70). Around 13% of the patients underwent unnecessary biopsies because of positive NBI.

Chen et al. improved the patient-level detection rate of NBI compared to that of WLI (97.9 vs. 88.8% *p* = 0.002). Tumor-level detection rates were 96.8 and 79.3%, respectively. False positive rates were not different in the NBI and WLI groups (76). Cauberg et al. reported a study wherein (77) each patient was evaluated by WLI and later by NBI in the same sitting by a different surgeon. NBI detected a mean of 2.1 lesions per patient, whereas WLI detected 1.7 lesions (*p* < 0.001). NBI had a detection rate of 94.7%, and WLI had 79.2%. The false positive rates of NBI were higher than those of WLI. NBI detected additional tumors in 35.9% of the patients. The detected additional tumors in this series were mainly grade 3 lesions.

Ye et al. reported their experiences in a multicentric randomized trial of WLI and NBI (78). The authors reported higher sensitivity with NBI and superior early bladder tumor and CIS detection (78). The specificity and false positive rates in the NBI arm were superior to those of the WLI examination but were not statistically significant (78). Giulianelli et al. in a comparative study, reported improved detection by the NBI arm by 30%, especially in patients with lesions smaller than 3 cm, and unifocal and recurrent lesions (79). Geavlete et al. reported on their experiences in a single institute study on within-patient comparison of WLI and NBI (80). Detection rates of NBI were better than those of WLI for papillary and CIS lesions. False positives were slightly higher in the NBI but not statistically significant. Moreover, NBI was useful in detecting positive tumor margins (80).

Tatsugami et al. (72) et al. also reported superiority of NBI-assisted diagnosis for detection of bladder tumors and CIS lesions, and in patients with positive and negative urine cytology findings. The reduced specificity of NBI in this series may be due to false detection of inflammatory lesions as cancerous by NBI. Zhu et al. reported a case series of twelve patients with positive or suspicious urine cytology (81). Patients in whom outpatient cystoscopy with WLI and imaging did not detect any bladder lesion were only included in the case series. Flexible cystoscopy was performed first with WLI and then with NBI. In this group of patients, sensitivity and specificity were 78 and 91% for NBI vs. 50 and 80% for WLI (81). In a single blind study on TURBT with NBI and with WLI from Korea, Kim et al. (82) reported better diagnostic yield with NBI than with WLI (85.5 vs. 80.9%). One-year recurrence-free rates were 85.2% with NBI and 72.2% with WLI. However, these recurrence-free rates were not significantly different (82).

Jecu et al. in a retrospective analysis, reported significantly improved detection rates of papillary NMIBC and CIS (94.9 vs. 88.1% and 95.7 vs. 65.2%) (83). NBI cystoscopy resulted in better diagnostic accuracy with NMIBC and CIS. Additional tumor detection rates for NBI were 56.6 vs. 8.7%, 28 vs. 10.3%, 30.3 vs. 10.6%, and 31.6 vs. 9.4% in patients with CIS, pTa, pT1, and NMIBC, respectively (83). One of the advantages of performing NBI at the end of an extensive TURBT for a tumor larger than 3 cm is the identification of infiltration of the bladder mucosa at the periphery of resection. Such infiltration is likely to be missed by WLI (84).

Drejer et al. investigated the effect of performing a flexible cystoscope with a high-definition (HD) camera system (85). The authors also evaluated the impact of NBI on the practical management of bladder cancer. Additional NBI findings were not considered clinically relevant. NBI changed the clinical decision in 1.9% of patients when HD cystoscopy was used as standard. However, HD cystoscopy also had high detection of non-malignant lesions (85).

It is common for NBI studies to study the bladder by WLI first and then by NBI (70, 72, 74). It can be argued that the better performance of NBI in these studies is the effect of the “second look” procedure, which adds to the performance of WLI, thereby improving the diagnostic efficacy of NBI. Moreover, most of the prior studies were concerned with the detection of recurrent lesions. This issue was addressed by Shen (86). In this single-center study, two highly experienced blinded observers performed cystoscopies with WLI or with NBI on a random imaging sequence (WLI followed by NBI and vice versa), thereby negating the effect of a second look. The study only enrolled primary suspected lesions that have not been biopsied or resected previously or have not received any intravesical therapy. In this controlled study, the sensitivity of NBI was significantly better than that of WLI, 92.9 and 77.7%, respectively. However, the specificity of NBI was lower than that of WLI, 73.5 and 82.7%, respectively. It is interesting to note that there were few lesions that were only detected by random biopsies and not by NBI or WLI. NBI was quite superior in detecting CIS. There were no CIS lesions that were detected by WLI and missed by NBI. Here, also, few CIS lesions that were detected by random biopsies were missed by both WLI and NBI. There has been a concern that most additional lesions detected by NBI are low-grade lesions. However, this is not the case (87).

Li et al. reported a meta-analysis of 1,040 patients across seven studies (88). This meta-analysis found NBI to be better than WLI in patient-level and tumor-level analyses. NBI detected lesions in an additional 17% of the patients and an additional 24% of the lesions (88). Zheng et al. reported similar improved detection rates on per-person analysis in their meta-analysis comprising 1,022 patients across eight studies (89). Another meta-analysis of twenty-five studies comprising 1,557 patients was published by Xiong et al. (90). NBI improved compared with WLI, with additional detection rates of 9.9 and 18.6% in per-patient and per lesion analyses. The additional detection rates in this meta-analysis were lower than those in the Li et al. meta-analysis (88) mentioned above.

In diagnosing bladder cancer, CIS is of particular importance. It is more often than not missed by WLI and optical enhancements come in as a handy tool for the detection of CIS. Most studies report data on CIS separately because of its potential risk of progression. Studies by Herr and Donat (70), Shen et al. (86), Tatsugami et al. (72), and Geavlete et al. (91) have all reported significantly improved detection of CIS lesions by NBI. A meta-analysis by Lee also reported improved detection of CIS lesions by NBI. NBI was able to detect an additional 28% CIS lesions. False positive rates between NBI and WLI were not significantly different (88). A meta-analysis by Xiong et al. (90) reported additional detection rates of 25.1% on per-patient and 31.1% on per-lesion analysis by NBI compared to WLI. Table 2 highlights the sensitivity and specificity of NBI and WLI.

**Table 2:** Characteristics of sensitivity and specificity of NBI and WLI.

**Reference**	**Detail**	**NBI sensy %**	**NBI Spey**	**NBI FPR**	**NBI PPV**	**NBI NPV**	**WLI Sens**	**WLI Spec**	**WLI FPR**	**WLI PPV**	**WLI NPV**
Ye et al. (78)	Pt level	97.7	50.0	50.0	91.4	80.0	66.67	25.0	75.0	82.86	12.12
Ye et al. (78)	Lesion level	98.8	60.90	39.10	76.04	97.59	75.45	58.65	41.35	69.61	65.55
Bryan et al. (74)		100					96.6				
Herr and Donat (70)		100	81.8				87.4	85.8			
Cauberg et al. (77)		94.7	68.4				79.2	75.5			
Tatsugami et al. (72)	BldT	92.7	70.9		63.4	94.7	57.3	86.2		69.2	78.8
Tatsugami et al. (72)	CIS	89.7	74.5		78.8	87.2	50.0	83.6		76.3	61.3
Shen et al. (86)		92.9	73.5				77.7	82.7			

#### Effect on Recurrence

Herr et al. reported their initial data on long-term follow-up of newly diagnosed low-grade, non-invasive papillary tumors (92). Of the original group of 215 patients, 126 were followed up by regular cystoscopies. These patients were later followed by WLI and NBI when NBI became available (93). Tumor recurrence rates for 3 years before and after NBI became available were compared. The authors reported fewer patients with recurrence, fewer number of recurrent lesions, and longer recurrence-free survival by NBI (93).

Performing TURBT entirely with NBI is feasible (94). Moreover, performing additional NBI-directed biopsies on patients with high-grade lesions at the end of extensive second TURBT leads to identification of missed high-grade lesions (84). A frequency-matched index control study on NBI vs. WLI TURBT was reported by Cauberg et al. (95). In the study group, all lesions detected by NBI or WLI were resected, and the control group was the retrospective matched group. Residual tumor rates at first follow-up cystoscopy were significantly less in NBI than in WLI (15 vs. 30.5% OR: 2.7, one-sided 95% CI: 1.2–6.1; *p* = 0.03) (95). Naselli et al. published their randomized trial of TURBT with WLI or with NBI (96). In this study, a complete procedure was performed with either WLI or NBI. Follow-up cystoscopies were also performed with same imaging modality. Patients were followed up for a period of 1 year. The bladder cancer detection rate of NBI was superior to that of WLI (1.55 vs. 1.36 lesions per person, *p* = 0.07). False positive rates were higher in NBI (28%) than in WLI (21%) but not significant. NBI reduced 1-year recurrence risk by at least 10% (96).

Naito et al. reported results of the Clinical Research Office of the Endourological Society (CROES) randomized trial of TURBT with WLI or with NBI (97). Patients with primary tumors were enrolled and randomized to TURBT with either WLI or NBI. Follow-up cystoscopies were performed with WLI. Surgery with NBI took a longer time. At 1-year follow-up, there was no difference in recurrence rates in the WLI or NBI group. Recurrence rates in low-risk lesions were, however, lower in the NBI group. But that was not the case with the intermediate and high-risk groups. With regard to the intermediate and high-risk groups, it is the biology of the tumors over which NBI does not have any control (97).

T1HG tumors have a poor risk of recurrence. Giulianelli et al. evaluated the use of NBI technology on resection of tumor margins and assessed the impact on tumor persistence in re-TURBT (98). Tumor persistence rate was 19%, which was lower than that of WLI. Moreover, none of their patients had pT2 disease in re-TURBT (98). Kobatake et al. reported a comparative study on NBI vs. WLI (99). Patients in respective groups underwent cystoscopy, TURBT, and follow-up cystoscopies with the same modality. No additional treatment was offered to these patients except for multiple biopsies and resection of recurrent lesions. NBI had higher sensitivity than WLI (95 vs. 70%; *P* < 0.01). Also, NPV was better with NBI (97.1 vs. 86.8%; *p* < 0.01). One-year recurrence rate with NBI was 21.1%, whereas with WLI it was 39.7% (*p* = 0.016), suggesting a beneficial effect of NBI on diagnosis as well as recurrence rates (99).

A meta-analysis by Xiong et al. (90) reported significantly reduced recurrence rates with NBI at 3 and 122 months. The outcomes of these trials make us believe that NBI TURBT has some role in delaying tumor recurrence (100). Kang et al. published a meta-analysis of RCTs performing NBI and WLI during TURBT (101). The authors reported significant benefit in recurrence rates at 3 months, 1, and 2 years. The risk ratios were 3-mo RR: 0.39 (95% CI, 0.26e0.60; *p* < 0.0001), 1-yr RR: 0.52 (95% CI, 0.40e0.67; *p* < 0.00001), and 2-yr RR: 0.6 (95% CI, 0.42e0.85; *p* = 0.004) compared with WLI TUR (101).

#### NBI After BCG Therapy

The reddish patches seen after intravesical BCG can be mistakenly considered as tumors. Switching to NBI, the suspicious neovascularized lesions look brownish and black, whereas lesions that look greenish with NBI are less likely to be tumors. Herr evaluated the utility of NBI in a post-BCG setting (102). In 21 out of 22 patients, NBI correctly identified a tumor. The false positive rate of NBI was 32% in this study. NBI outperformed urine cytology in detecting patients with persistent lesions. This would have a great prognostic implication in this high-risk group of patients in identifying patients who would be candidates for more intensive therapy (102). The results published by Herr (102) were, however, not replicated by other researchers. It has been argued that NBI in a post-BCG setting may lead to more unnecessary biopsies, especially if the duration between last BCG instillation and cystoscopy is shorter (103). In another review, Herr proposed deferring a 3-month biopsy in post-BCG patients with negative cytology and benign-looking lesions (104).

#### Therapeutic Impact of NBI and Effect on the Progression

It is proposed that better visualization, improved staging, better local control, and fewer recurrences with NBI would translate to an improved therapeutic impact of NBI (104). Despite this, its effect on the meaningful endpoint is unknown (78). Shadpour et al. applied the EORTC scoring system to tumors detected by WLI and NBI in the same cohort of patients. Progression risk scores were not statistically different between NBI and WLI (87). More prospective studies are needed to evaluate the beneficial effect of NBI on the ultimate therapeutic outcome (104).

#### Cost-Effectiveness of NBI

There are limited data on the cost-effectiveness of NBI. In one review, NBI resulted in estimated savings of 230-500 USD per year; probably a result of the reduced recurrence rates. However, they excluded the cost of pathological analysis and prolonged operation times (105).

#### Narrow-Band Imaging vs. Photodynamic Diagnosis

In contrast to PDD,which depends on stronger absorption and extended excretion of protoporphyrin by cancer cells rather than normal bladder tissues; NBI is not cancer-specific. It depends on the improvement of the morphological visibility of the superficial epithelium (106). Interpretation of NBI is subjective.

A recently published meta-analysis including 4,519 patients by Motlagh et al. that focused on enhancement techniques during TURBT in NMIBC found that performing PDD during TURBT with concurrent single immediate intravesical chemotherapy (SIIC) resulted in superior recurrence outcomes. A significant reduction in 12- month recurrence rate performing PDD along with an increase in risk benefit by an additional 32% reduction in odds ratio was noticed when using concomitant SIIC (107).

Naya et al. were the first to report a comparative study on PDD with oral 5-ALA and NBI simultaneously in the same patients (108). In this study, 10 patients were included. All underwent WLI cystoscopy first, followed by NBI, and then PDD in an alternating sequence. Patients with abnormal cytology and undefined papillary mucosa were recruited. The results were more in favor of PDD. PDD detected all cancer lesions and missed 4% of dysplasia. NBI missed 5% of CIS and 10% of dysplasia. Sensitivity for Cis and dysplasia detection was 91.6% for PDD and 62.5% for NBI (108). Another larger study reported by Drejer et al. recruited 171 patients (109). Patients underwent cystoscopy with WLI followed by NBI and PDD. In the patient-level analysis, compared to WLI, NBI and PDD showed a significantly higher sensitivity for detection of CIS and dysplasia. The sensitivities were NBI 95.7%, PDD 95.7%, and WLI 65.2% (*p* < 0.05). But the specificities were not statistically different (NBI: 52, PDD 48, and WL 56.8%). On per biopsy analysis, NBI and PDD both had better sensitivity than WLI. Per lesion sensitivities were 72.2, 78.2, and 52.7% for NBI, PDD, and WLI, respectively (*p* < 0.05). PPVs were not different among these modalities (NBI 23.7, PDD 22.2, and WL 19%). NBI was suggested as a valid alternative to PDD by these authors (109).

Kwon et al. published a network meta-analysis of therapeutic outcomes of TURBT guided by WLI, NBI, PDD ALA, and PDD HAL (110). The authors found that the recurrence rate of TURBT with 5 ALA was lower than that of TURBT with HAL guide (OR = 0.48, 95 % CI 0.26–0.95). Theoretically, HAL has better penetration and accumulation in neoplastic cells, but 5-ALA has been evaluated in more studies in this meta-analysis. This might be the reason for the superiority of 5ALA over HAL in this meta-analysis. Recurrence rates with ALA- and NBI-guided TURBT were similar. All three imaging modalities, i.e., ALA, HAL, NBI, had lower recurrence rates than WLI TURBT. But there were no significant differences in progression rates (110). NBI had the highest rank probability for progression-free rate. The authors also suggested that NBI would be preferable in patients who have undergone intravesical instillations (110).

Chen et al. published a meta-analysis of 26 studies including 3,979 patients. The studies used WLI as the index imaging modality and PDD with HAL or 5-ALA or NBI as the comparator imaging modality (111). In the lesion-level analysis, pooled diagnostic odds ratio (DOR) was highest with HAL at 78.14 (95% CI 31.42–194.28), followed by NBI at 40.09 (95% CI 20.08–80.01). DOR was lowest with 5-ALA at 18.14 (95% CI 4.28–76.87). Higher DOR indicates better performance of the test, and HAL performed better here. Area under the receiver operating curve (AUROC) was, again, best with HAL (0.94) (95% CI 0.92–0.96) followed by NBI (0.88) (95% CI 0.85–0.91), and 5ALA (0.82) (95% CI 0.79–0.85). Pooled sensitivities of HAL, NBI, and 5-ALA were 0.95, 0.94, and 0.9, respectively. Specificities were 0.81, 0.79, and 0.69, respectively. In the patient-level analysis, NBI had the best DOR at 358.71 (95% CI 44.5 to 2,891.71) followed by HAL at 59.95 (95% CI 24.3–147.92), and 5-ALA at 79.52 (95% CI 0.94–6,759.92). The authors proposed that NBI could be the most promising diagnostic modality for NMIBC looking at cost, reliability, and simplicity (111).

#### Current Place

Narrow-band imaging significantly improves bladder cancer management with improved detection rates and reduced recurrence rates. The lower recurrence rates are irrespective of intravesical instillation (112). It is likely that it would lead to more relaxed surveillance. It is easy to learn and cost-effective. Considering cost, reliability, and simplicity, it may be a promising modality (111). However, it is not as extensively studied as PDD.

### Image 1 S

#### Technical Consideration

In image 1S, images are processed on a software platform to give different contrast specifications. Different light wavelengths are used to produce images with different contrast specifications. A white light image is enhanced by two modalities, Clara and Chroma. Clara enhancement creates a clearer image of darker regions by local brightness adaptation. Chroma enhancement improves the sharpness of an image by enhancing local color contrast. These modes can be used together as Clara + Chroma. In addition to this, there are two predefined post-processing virtual chromoendoscopy enhancements called spectra A (SA) and spectra B (SB). In these SA and SB modes, different color contrast is generated by changing the effective spectral response in an image (113). The original WLI image and the enhanced mode are shown in the endoscopy monitor side by side in real time, in contradistinction with PDD or NBI (114).

In the spectra A (SA) mode, the green and blue light signals from RGB signals are separated. The contrast of capillaries and vessels in the superficial mucosa and submucosa is highlighted in the SA mode. In the spectra B (SB) mode, there is no color transformation, but a color tone shift occurs. In this mode, in addition to the vessels in superficial mucosa and submucosa (which are highlighted in SA), deeper tissue layers are also visible (113). Using SB may be beneficial in case of visual interferences because of hematuria (11).

Kamphuis et al. evaluated the image enhancement capabilities of SA and SB under three different layer thickness values of 100, 200, and 300 um. For superficial layers, both SA and SB showed increased absorbance, whereas for intermediate layers SA and SB both showed more absorbance than white light. Absorbance was significantly higher with the SB mode in the intermediate layer. For deeper layers, both white light and SB were more sensitive to absorbance than SA. The authors hypothesized that SA and SB would help in the differentiation of normal mucosa and tumors (113).

Kamphuis et al. evaluated differences in interpretation of the bladder urothelium imaged with image 1S enhancement modes on iPad app (115). Cystoscopy images were recorded in WLI, SA, and SB, as well as Clara and chroma combined modes. They selected 20 bladder areas for the study, thereby giving 80 images. These images were shown to 73 participants on iPad. The participants were asked to delineate abnormal-looking areas with a stylus. Instead of histological diagnosis, the assessment of images by a panel of urologists at the Academic Medical Center, Amsterdam, the Netherlands was used as the control. The panel classified these images as easy to delineate (agreement) and difficult to delineate (disagreement). Observers graded the quality of images with image 1 S enhancement to be better than WLI. In this study, the authors observed less variation in interpretation in chroma + clara and SB than WLI and SA in easy to delineate cases. The quality of images in enhancement modes was graded significantly better by the participants (115).

#### Learning Curve

Soria et al. presented a conference study evaluating the effect of a surgeon's experience on the correct cystoscopic diagnosis of bladder cancer by WLI, PDD, and image 1S (116). Twenty-six patients with prior history of high-grade bladder cancers or positive urine cytology and negative ultrasound were included. Cystoscopies were performed by a senior urologist with long-term experience in PDD. Video recordings of these cystoscopy procedures were shown to urology residents not experienced in PDD or image 1S, and they were asked to map areas where they would take a biopsy. The residents' remarks were compared with the procedures performed by experienced urologists. A lower rate of missed bladder cancer lesions by PDD as well as image 1S irrespective of the experience of the surgeons was noted. The authors concluded that both PDD and image 1S would help in decreasing the chance of false negative cystoscopies even with less experienced observers. In this study, the interobserver agreement rate did not differ among WLI, image 1S, and PDD (116).

#### Role in Diagnosis

Chondros et al. presented the preliminary results of their ongoing study on image 1S in a conference article (117). In this study, each patient underwent cystoscopy with WLI and the SB mode with random allocation by two independent experienced urologists. Enhanced cystoscopy identified significantly (*p* = 0.003) more (48/78, 61.5%) lesions than WLI (37/78, 47.8%). The advantages of image 1 S were better viewing experience and better identification of suspicious lesions. This method does allow for a within-patient comparison of WLI and S enhancement modes. There was a better correlation of positive urine cytology with SB mode findings but not with WLI. The SB mode enhancement was found to be superior to WLI in the follow-up setting (117).

Soria et al. reported their experience in bladder cancer diagnosis of patients with prior history of high-grade bladder cancers or positive urine cytology and negative ultrasound (116). Compared with WLI, image 1 had a lower rate of missed lesions. The overall concordance rate was also better in image 1S than in WLI. This study, however, neither compared the performance of image 1S or PDD with that of histological diagnosis, nor did the abstract mention which of the image enhancements was used in the recordings (116). Mulawkar et al. published their findings of a web-based survey on cystoscopy images in WLI and SA and SB modes (118). Cystoscopy images from patients with known or suspected bladder cancer and some patients with recurrent urinary infections, invisible hematuria, and storage or voiding symptoms were included. Side-by-side cystoscopy images were cut into two parts, with the first part being WLI and the second being SA or SB. A total of 10,786 observations were included in the analysis. In this study, the side-by-side images were cut into two parts, WLI and SA or SB. The individual images were shown to observers in a random fashion. In patients without cancer, SA or SB enhancement did not add much to the diagnostic accuracy of WLI. But in patients with cancer, SA and SB added significantly to the diagnostic accuracy of WLI. Negative SA and SB ruled out bladder cancer more effectively than WLI (118).

#### Comparison of Image 1S With Other Macroscopic Enhancement Modalities

Soria et al. presented their study comparing the performance of WLI, PDD, and image 1S (116). Both PDD and image 1S performed better than WLI and were comparable with each other. But this study was not designed to compare PDD with image 1S head-to-head (116).

#### Effect on Recurrence, Progression

The Clinical Research Office of the Endourological Society (CROES) is currently conducting a multicenter randomized control trial of WLI and image 1 S (119). In this study, the control arm is WLI cystoscopy-assisted TURBT and the study arm is WLI + image 1S-assisted TURBT. Here, randomization is stratified by a multiplicity of tumors, the primary or recurrent status of the tumors, and macroscopic appearance. The authors aim to study recurrence rate and perioperative morbidity. It is postulated that an enhanced image may help the demarcation between a tumor and a healthy tissue, leading to reduced recurrence rates. The results of this study are awaited (119).

#### Possible Advantages of Image 1S Over NBI and PDD

Lapini et al. published a comparative observational study on PDD vs. WLI (120). In this study, each patient was examined initially by white light and after that by blue light (PDD) cystoscopy. Then, biopsies of the lesions were performed. All cystoscopies were performed by one endourologist. This is a within-patient comparison of both diagnostic modalities. As the same examiner does WLI as well as PDD cystoscopy, this prolongs operative time, and such an evaluation is subject to “second look bias.” Geavlete et al. reported a prospective randomized study on PDD and WLI (38). The authors intended to evaluate the impact of PDD on diagnostic accuracy and treatment changes in NMIBC. Herein, one arm underwent standard WLI cystoscopy and transurethral resection of bladder tumor (TURBT). The study arm underwent standard WLI cystoscopy as well as PDD cystoscopy. The lesions detected on WLI underwent standard WLI cystoscopic resection. The lesions detected on PDD only underwent PDD-guided resection. This was followed by a PDD-guided assessment of resection margins. In this trial, the control and study group patients are different.

Stenzl et al. reported a multicenter prospective randomized double blind trial of PDD (49). The study group underwent standard PDD cystoscopy and TURBT. The control group underwent intravesical instillation of sodium chloride instead of 5-ALA. The patients in both groups were comparable but not the same. If a urologist is aware that a better image enhancement modality like PDD is available for bladder inspection after WLI cystoscopy, a bladder examination by WLI might be performed just superficially (42).

The image 1S system would have advantages over the conventional PDD system. In image 1S, there is no need to instill any medication in the bladder prior to cystoscopy with HAL or ALA, a solution must be freshly prepared. This does increase the time a patient remains in a hospital and would increase costs. Intravesical instillation of 5-ALA or HAL is quite safe, but there has been a report of anaphylactic shock attributed to intravesical instillation of hexvix (HAL) (21). The shock manifested 5 h after instillation. It has been proposed to be a non-immunoglobulin E-mediated allergic reaction. In this case, the dwell time of HAL was 3 h. No bladder tumor was detected, and the patient underwent resection of the prostate. Serum tryptase was raised, and skin test for HAL allergy was positive; hence, there is enough reason to believe that anaphylaxis to HAL is possible.

Photodynamic diagnosis also needs procurement of a new camera, telescope, and light carrier. This might be a good proposition for a busy unit but would not be worth the investment if additional equipment in image 1S and PDD is equivalent. There is some time lag in switching from WLI to PDD. In image 1S, both images are viewed on the same screen side by side. This is helpful for the comparison of different features. The quality of video images and motion artifacts (121) with PDD are also worth considering. However, as of today, we do not have robust studies comparing PDD to image 1S.

Herr and Donat published their study on reduced bladder tumor recurrence rates with NBI in patients on surveillance cystoscopy (93). WLI and NBI cystoscopy were done by the same observer. In the initial part of the study, WLI was performed, and later when NBI became available, both WLI and NBI cystoscopy were performed. Here, the patient acted as his own control. The main objection to this study is the potential “second look bias.” Naselli et al. conducted a randomized controlled trial of TURBT with WLI and NBI (96). In one group, TUR was performed with WLI and in the other with NBI entirely. Switch from WLI to NBI or vice versa was not allowed. A second TUR, if required, was also performed in the same modality. This study addresses the utility of image enhancement in reducing recurrence rates. But this study does not tell us about the “within patient” performance of NBI (96).

Cauberg et al. reported on a prospective trial of NBI vs. WLI (77). In this trial, WLI and NBI cystoscopy of the same patient was performed by two independent observers separately in the same sitting. The NBI observer was blinded to the WLI findings. This technique allowed for a “within-patient” comparison of WLI and NBI. It also eliminated the second look bias but at the cost of prolonging the operative time of cystoscopy and anesthesia for the cystoscopy.

There are some theoretical advantages in using image 1S rather than NBI. NBI requires a special light source, whereas image 1S does not need any such investment. While examining the bladder by image 1S, there is no extra time taken to change the light source from WLI to NBI mode. With image 1S, as both the WLI and spectra images are viewed on the same screen side by side, one gets a real-time perception of the extent of a tumor compared to NBI where the old image is not available for comparison after switching to NBI from WLI. This would theoretically help in the proper completion of TURBT. Operative time is saved, and no extra anesthesia time is needed, thereby reducing the risk of the procedure, even if minimal. However, to conclude whether NBI or image 1S is better, more data are needed. If spectra modes in image 1S are reliable and reproducible in the diagnosis of bladder lesions, these modes do have the potential of replacing NBI and PDD.

#### Current Place

Image 1S enhancement modalities need comprehensive evaluation under actual clinical conditions. Its beneficial role in the diagnosis of bladder cancer is promising. Its exact accuracy, sensitivity, and specificity in a clinical scenario need to be studied. The role of image 1S in the recurrence and progression of bladder cancer is not yet clear. Its exact place *vis a vis* other established modalities like PDD and NBI needs further trials. If not inferior or equivalent to PDD and NBI, it has the potential of replacing these two modalities for the reasons explained above.

### Other Emerging Macroscopic Imaging Modalities

Emerging macroscopic techniques are twin monitor-mode PDD, autofluorescence, and scanning fiber endoscope.

A possible shortcoming of PDD is the ability to view only one image at a time and time taken for switching to blue light and generation of an image, thereby losing the reference WLI image. In a comparative study, Fukuhara et al. tried to overcome this issue by side-by-side visualization of reference WLI image and PDD image in real time (122). They used a flexible bronchoscope (off label use) for this purpose. In this system, white light is emitted in the first 1/60 s followed by blue light in the next 1/60 s. These two images are held in a memory chip, and each image is displayed for 1/30 s on a monitor at a frame rate of 30 per s. This system was compared with the conventional PDD system in terms of better sensitivity, specificity, and reduce false positive rates. Further studies on this novel technique are awaited (122).

Schäfauer et al. developed the first prototype for bladder cancer detection based on the principle of ultraviolet autofluorescence (123). This uses endogenous fluorophores in tissues, which are excited by ultraviolet light, and autofluorescence is measured and color-coded. The prototype was able to differentiate between normal urothelium and bladder cancer (123). Kriegmair et al. reported their pilot study on wide-field autofluorescence-guided TURBT (124). In this pilot study, the authors used a D-Light system and customized a band pass filter at the eyepiece of the endoscope. No intravesical instillation is required. Normal urothelium looks greenish, and papillary tumors look brown-reddish. This technique has the potential to increase detection rates of bladder cancer (124).

Scanning fiber endoscope uses an ultrathin endoscope. This can be used as a standalone endoscope or as a probe (125). It can be steered remotely, and images generated from it can be stitched to generate a 3D mode of the urinary bladder (126).

## Conclusions

All these macroscopic image enhancement modalities have proven their utility in improved detection and short-term cancer control. Some studies have shown beneficial effects in terms of delayed progression and improved post cystectomy outcomes with PDD. NBI may be an acceptable alternative to PDD if some of the meta-analyses are to be believed. Most of the image enhancement modalities have not proven their utility in delaying progression or long-term cancer control. Most of these modalities claim to result in more complete TURBT. The presence of detrusor muscle is the surrogate marker for the completeness of TURBT. Data on the effect of an image enhancement modality on the presence of detrusor muscle in TURBT specimens are lacking. Cancer progression and long-term control are governed by the biological nature of cancer cells. PDD may have some role in early detection in this regard. However, well-designed studies are needed to establish the efficacy of these modalities in the evaluation of patients with bladder cancer. The last word in this regard is yet to be written.

## Author Contributions

All authors listed have made a substantial, direct, and intellectual contribution to the work and approved it for publication.

## Conflict of Interest

GS was employed by Chitale Clinics Private Ltd. The remaining authors declare that the research was conducted in the absence of any commercial or financial relationships that could be construed as a potential conflict of interest.

## Publisher's Note

All claims expressed in this article are solely those of the authors and do not necessarily represent those of their affiliated organizations, or those of the publisher, the editors and the reviewers. Any product that may be evaluated in this article, or claim that may be made by its manufacturer, is not guaranteed or endorsed by the publisher.
